# A human ribonuclease induces apoptosis associated with p21^WAF1/CIP1 ^induction and JNK inactivation

**DOI:** 10.1186/1471-2407-11-9

**Published:** 2011-01-11

**Authors:** Jessica Castro, Marc Ribó, Susanna Navarro, Maria Victòria Nogués, Maria Vilanova, Antoni Benito

**Affiliations:** 1 Laboratori d'Enginyeria de Proteïnes, Departament de Biologia, Facultat de Ciències, Universitat de Girona, Campus de Montilivi s/n E-17071 Girona, Spain; 2 Departament de Bioquímica i Biologia Molecular, Facultat de Biociències, Universitat Autònoma de Barcelona, 08193 Bellaterra, Spain

## Abstract

**Background:**

Ribonucleases are promising agents for use in anticancer therapy. Among the different ribonucleases described to be cytotoxic, a paradigmatic example is onconase which manifests cytotoxic and cytostatic effects, presents synergism with several kinds of anticancer drugs and is currently in phase II/III of its clinical trial as an anticancer drug against different types of cancer. The mechanism of cytotoxicity of PE5, a variant of human pancreatic ribonuclease carrying a nuclear localization signal, has been investigated and compared to that of onconase.

**Methods:**

Cytotoxicity was measured by the MTT method and by the tripan blue exclusion assay. Apoptosis was assessed by flow cytometry, caspase enzymatic detection and confocal microscopy. Cell cycle phase analysis was performed by flow cytometry. The expression of different proteins was analyzed by western blot.

**Results:**

We show that the cytotoxicity of PE5 is produced through apoptosis, that it does not require the proapoptotic activity of p53 and is not prevented by the multiple drug resistance phenotype. We also show that PE5 and onconase induce cell death at the same extent although the latter is also able to arrest the cell growth. We have compared the cytotoxic effects of both ribonucleases in the NCI/ADR-RES cell line by measuring their effects on the cell cycle, on the activation of different caspases and on the expression of different apoptosis- and cell cycle-related proteins. PE5 increases the number of cells in S and G_2_/M cell cycle phases, which is accompanied by the increased expression of cyclin E and p21^WAF1/CIP1 ^together with the underphosphorylation of p46 forms of JNK. Citotoxicity of onconase in this cell line does not alter the cell cycle phase distribution and it is accompanied by a decreased expression of XIAP

**Conclusions:**

We conclude that PE5 kills the cells through apoptosis associated with the p21^WAF1/CIP1 ^induction and the inactivation of JNK. This mechanism is significantly different from that found for onconase.

## Background

Members of the pancreatic ribonuclease (RNase) superfamily display an array of biological activities ranging from cytotoxicity to angiogenesis. Among them, cytotoxicity is one of the most attractive since such enzymes could be used, alone or conjugated to ligands or antibodies, as non-mutagenic therapeutic agents for cancer treatment (for a review, see [1]). Among the different natural or engineered RNases described to be cytotoxic, the case of onconase, a monomeric RNase isolated from *Rana pipiens *(northern leopard frog), is paradigmatic. This drug manifests cytotoxic and cytostatic effects [2], presents synergism with several kinds of anticancer drugs (for a review, see [3]), and is currently in phase II/III of clinical trial as an anticancer drug against different types of cancer [4]. Ribonucleolytic activity of onconase, exerted *in vivo *in the cytoplasm, is essential for its cytotoxicity [5]. Its natural substrates are t-RNA and most probably the non-coding RNA (microRNA) that is involved in regulation of gene expression through RNA interference (for a review, see [3]). Indeed, it has been postulated that the observed synergisms of this RNase may be a consequence of onconase targeting specifically the family of microRNA shown to enhance tumor resistance to cytotoxic anticancer therapy by mobilizing the cell defense mechanisms [6]. Onconase, nevertheless, shows renal toxicity at high concentrations [7,8]. Generating a cytotoxic variant of a human RNase such as the human pancreatic RNase (HP-RNase) would undoubtedly provide a potentially useful therapeutic agent which would be expected to have lower immunogenicity and renal toxicity than onconase.

Several variants of HP-RNase have been described as cytotoxic (for a review see [1]) and most of them are resistant to the cytosolic RNase inhibitor (RI). HP-RNase does not show renal accumulation *in vivo *[7,8] and presents around 10^3^-10^4 ^times higher ribonucleolytic activity compared to onconase [9]. We have reported a cytotoxic variant of HP-RNase, named PE5, which, despite being sensitive to the RI, is cytotoxic for a panel of diverse cell lines [10]. Nuclear import assays showed that PE5 was efficiently transported to the nucleus where it was initially localized in the nucleolus. The variant carries a conformational bipartite nuclear localization signal [11] which targets the protein to the nucleus, specifically in the nucleolus [10], where RI is absent, therefore producing the degradation of nuclear RNA [12].

In the present work we have observed that one of the cell lines most sensitive to the action of PE5 is NCI/ADR-RES. This ovarian cancer cell line is representative of poor-prognosis ovarian cancer. It is worth mentioning that ovarian carcinoma is the deadliest gynecologic malignancy and the fifth leading cause of death due to cancer in women in the United States [13]. Because ovarian cancer is often diagnosed only after the disease has reached an advanced stage, the majority of patients require additional treatment after surgical removal of the tumor. Aggressive surgical debulking and platinum plus taxane therapy have improved median survival from one year in 1975 to approximately five years in 2005, but the long-term cure rate continues to languish in the 20% to 30% range [14] because most cases ultimately develop resistance. Approximately 50% of the patients will enter a first complete clinical remission, yet 90% of suboptimally debulked patients and 70% of optimally debulked patients relapse in 18 to 24 months. For this reason, novel approaches are being sought to overcome chemoresistance and develop more effective therapies. Currently, biological therapies are being considered as the next approach in the fight against ovarian cancer. These therapies have the potential to selectively target tumors, to minimize toxicity, and to overcome the resistance often observed with conventional therapies [15].

Since PE5 is directed to the nucleus where it degrades nuclear RNA [12] while onconase displays its activity degrading cytosolic RNA, we sought to compare the cytotoxic properties of both RNases on this NCI/ADR-RES cell line. We also wanted to check whether this RNase could be a candidate to have its antitumor properties evaluated *in vivo*. For this, we were interested in investigating the selectivity of PE5 against tumor cells lines and to determine whether this RNase kills the cells through induction of apoptosis.

## Methods

### Ribonuclease expression and purification

Construction of plasmids expressing onconase (pONC) and PE5 (pPE5) have been previously described [10,16,17]. PE5 was constructed from PM5 replacing G89 and S90 by arginine [10]. PM5 codes for HP-RNase and incorporates the substitutions R4A K6A Q9E D16G and S17N [17]. Recombinant onconase and PE5 were produced and purified from *E. coli *BL21 (DE3) cells transformed with the corresponding vector essentially as described previously [18,19]. Briefly, frozen pellets from 2 L of induced culture were resuspended in 30 ml of 50 mM Tris-HCl, (pH 8.0), 10 mM EDTA. Cells were lysed using a French press and inclusion bodies were harvested by centrifugation. Pellets were then resuspended in 10 ml of 6 M guanidinium-HCl, 10 mM EDTA, 50 mM Tris-acetate, (pH 8.0). Reduced glutathione was added to a final concentration of 0.1 M, the pH was adjusted to 8.5 with solid Tris, and the samples were incubated at room temperature for 2 h under nitrogen atmosphere to assist protein solubilization. Insoluble material was removed by centrifugation and solubilized protein was diluted dropwise into 0.5 M L-arginine, 1 mM oxidized glutathione, 2 mM EDTA, 0.1 M Tris-acetate, (pH 8.5), and incubated at 4°C for at least 24 h. To stop oxidation, the pH was then lowered to 5 with acetic acid and the proteins were concentrated by ultrafiltration using a Prep/Scale TFF cartridge (Millipore, Bedford, MA, USA). In the case of onconase, cyclization of the N-terminal Gln residue to pyroglutamic acid is essential for its full catalytic activity and its cytotoxic properties. This is accomplished at this step by dialysis against 200 mM sodium phosphate (pH 7.2) at room temperature for a period of 48 h [19]. Both onconase and HP-RNase variants were then dialyzed against 50 mM sodium acetate (pH 5). Precipitated or insoluble material was eliminated by centrifugation, and the sample was loaded onto a Mono-S HR 5/5 FPLC column (Amersham Biosciences, Piscataway, NJ, USA). Fractions containing pure RNases were dialyzed against water, lyophilized and stored at -20°C. A yield of 15-20 mg of protein per 1 L of culture was obtained. The molecular mass of each variant was confirmed by matrix-assisted laser desorption/ionization time-of-flight mass spectrometry in the "*Unitat cientificotècnica de suport*" of the *Institut de Recerca *of the *Hospital Universitari Vall d'Hebron *(Barcelona, Spain). The protein concentration of each variant was determined by UV spectroscopy using an extinction coefficient of ε_278 _= 7950 M^-1 ^cm^-1 ^for PE5 and of ε_278 _= 10470 M^-1 ^cm^-1 ^for onconase, calculated using the method devised by Pace et al. [20].

### Cell lines and culture conditions

NCI/ADR-RES ovarian cancer cells (formerly MCF-7/Adr) were a generous gift from *Institut Català d'Oncologia de Girona, Hospital Universitari de Girona Dr. Josep Trueta *(Girona, Spain); HeLa cervical cancer cells and normal human fibroblasts N1 were obtained from Eucellbank (*Universitat de Barcelona*, Barcelona, Spain). Cells were routinely grown in Dulbecco's modified Eagle's medium (Gibco, Berlin, Germany) supplemented with 10% fetal bovine serum (FBS, Gibco, Berlin, Germany), 50 U/ml penicillin and 50 μg/ml streptomycin (Gibco, Berlin, Germany) and were maintained at 37°C in a humidified atmosphere of 5% CO_2_. NCI/ADR-RES cells were maintained in a complete Dulbecco's modified Eagle's medium containing 1 μg/ml doxorubicin (Tedec-Meijic Farma, Madrid, Spain). Cells remained free of *Mycoplasma *and were propagated in adherent culture according to established protocols.

### Cell proliferation assay

Cells were seeded into 96-well plates at the appropriate density, i. e., 7000 (for NCI/ADR-RES), 2200 (for HeLa) and 3000 (for N1) cells. After 24 h incubation, cells were treated with various concentrations of PE5 (0,1-30 μM) or onconase (0,001-10 μM) for 72 h. In separate experiments, cells were treated with SP600125 (0.1-80 μM diluted in dimethyl sulfoxide (DMSO)) or DMSO alone for 96 h. Media with either SP600125 or vehicle were replaced every 36 h. Drug sensitivity was determined by the MTT method essentially as described by the manufacturer's instructions (Sigma, St. Louis, MO, USA). Data were collected by reading at 570 nm with a multi-well plate reader (Anthos Labtec, Cambridge, UK). The IC_50 _value represents the concentration of the assayed enzyme required to inhibit cell proliferation by 50% respective to cells treated with the carrier and in each case was calculated by linear interpolation from growth curves obtained.

### Analysis of cytostatic and cytotoxic effects of PE5 and onconase

NCI/ADR-RES cells (55000 per well) were seeded into 24-well plates and then treated with different concentrations of PE5 (2-35 μM) or onconase (0.3-5 μM) for 24, 48 and 72 h. After treatment, attached and floating cells were harvested and washed in cold phosphate buffered saline (PBS). The proliferation rate and viability in control and RNase-treated cultures was estimated by cell count using a hemocytometer combined with the trypan blue exclusion assay.

### Cell cycle phase analysis

Cell cycle phase analysis was performed by propidium iodide (PI) staining. NCI/ADR-RES cells (1.2 × 10^6 ^cells/100-mm dish) were treated with 35 μM PE5 or 1 and 5 μM onconase for 72 h. Attached and floating cells were then harvested and fixed with 70% ethanol for at least 1 h at -20°C. Fixed cells were harvested by centrifugation and washed in cold PBS. These collected cells were resuspended in PBS (1-2 × 10^6^/ml) and treated with RNase A (100 μg/ml) and PI (40 μg/ml) (Molecular Probes, Eugene, OR, USA) at 37°C for 30 min prior to flow cytometric analysis. A minimum of 10,000 cells within the gated region were analyzed on a FACSCalibur flow cytometer (BD Biosciences, San Jose, CA, USA). Cell cycle distribution was analyzed using the FlowJo program (FreeStar, Ashland, OR, USA).

### Apoptotic morphology analysis

Experiments were carried out in serum-starved medium, at 37°C and in a 5% CO_2 _atmosphere. NCI/ADR-RES cells (2.2 × 10^3 ^per well) were treated with 35 μM PE5 or 5 μM onconase for 72 h followed by the addition of 4 μM calcein AM and 20 μg/ml PI (Molecular Probes, Eugene, OR, USA). The effect of PE5 and onconase was analyzed using a laser scanning confocal microscope (Leica, Wetzlar, Germany). Images of cells stained with calcein AM and PI were acquired using a 488-nm argon laser and a 561-nm HeNe laser, respectively.

NCI/ADR-RES cells (2.2 × 10^4 ^per well) were also seeded in 35-cm^2 ^plates with a glass coverslip and treated with 35 μM PE5 or 5 μM onconase. After 72 h cells were incubated with 0.5 μg/ml Hoechst 33342 (Molecular Probes, Eugene, OR, USA) for 30 min at 37°C in the dark. Confocal images of the cultures were captured using a laser scanning confocal microscope and a 405-nm blue diode laser.

### Phosphatidylserine exposure assay

Quantitative analysis of apoptotic cell death caused by PE5 and onconase treatment was performed by flow cytometry using the Alexa Fluor 488 annexin V/PI Vybrant Apoptosis Assay Kit (Molecular Probes, Eugene, OR, USA) following the manufacturer's instructions. Briefly, NCI/ADR-RES cells (2.2 × 10^5 ^per well) were seeded into 6-well plates and then treated with 35 μM PE5 or 5 μM onconase for 24, 48 and 72 h in serum-starved medium. After treatment, attached and floating cells were harvested, washed in cold PBS and subjected to Annexin V-Alexa Fluor 488 and PI staining in binding buffer at room temperature for 15 min in the dark. Stained cells were analyzed on a FACSCalibur flow cytometer using CellQuest Pro software. A minimum of 10,000 cells within the gated region were analyzed.

### Caspase activation assay

Caspase-3, -8 and -9 catalytic activities were measured using the APOPCYTO Caspase-3, -8 and -9 colorimetric assay kits (MBL, Nagoya, Japan) following the manufacturer's protocol. The assay is based on cleavage of the chromogenic substrates, DEVD-pNA, IETD-pNA and LEHD-pNA, by caspases-3, -8 and -9, respectively. Briefly, NCI/ADR cells (1.2 × 10^6 ^cells/100-mm dish) were incubated with 35 μM PE5 or 5 μM onconase for 24, 48 and 72 h in serum-starved medium. Then, attached and floating cells were lysed and centrifuged. The supernatant was recovered, and the protein concentration was determined using the Bradford protein assay (Bio-Rad Laboratories, Hercules, CA, USA). Afterwards, 50 μl of the cell lysate corresponding to 110 μg of total protein, 50 μl of 2X reaction buffer containing 10 mM DTT and 5 μl of the 10 mM DEVD-*p*NA, IETD-*p*NA or LEHD-*p*NA substrates were added to each well of the 96-well plates. To confirm the specific hydrolysis of substrate, samples were treated with the caspase inhibitors DEVD-FMK, IETD-FMK or LEHD-FMK, which are specific inhibitors of caspases-3, -8 and -9, respectively, following the manufacturer's protocol. Then the plate was incubated at 37°C for 4 hours. The reaction was measured by changes in absorbance at 405 nm.

### Western blot analysis

NCI/ADR-RES cells (1.2 × 10^6 ^cells/100-mm dish) were incubated with 7 μM PE5 or 1 and 5 μM onconase for 72 h, harvested with trypsin-EDTA solution and washed in cold PBS. Cells were lysed in lysis buffer (Cell Signalling Technology, Beverly, MD, USA) and a sample was taken to measure protein content using the Bradford protein assay. Protein samples were separated on a 12.5% SDS-PAGE gel and transferred to polyvinylidene difluoride membranes (Millipore, Bedford, MA, USA). The membranes were incubated for 1 h at room temperature in blocking buffer [3% powdered-skim milk in TBS-T (10 mM Tris-HCl (pH 7.5), 100 mM NaCl and 0.1% Tween-20)] to prevent non-specific antibody binding. Antibody dilution was prepared in blocking buffer and blots were incubated with monoclonal antibody overnight at 4°C. Antibodies against Bcl-2 (# sc-7382, 1:200 dilution), Bax (# sc-7480, 1:1000 dilution), Cyclin D1 (# sc-20044, 1:1000 dilution), Cyclin E (# sc-247, 1:1000 dilution), p27 (# sc-1641, 1:200 dilution), JNK (# sc-7345, 1:50 dilution), p-JNK (# sc-6254, 1:1000 dilution) were from Santa Cruz Biotechnology (Santa Cruz, CA, USA). The antibodies against JNK and phosphorylated JNK recognize JNK1, JNK2 and JNK3; however JNK3 is not expressed in ovarian cells. Antibodies against GAPDH (Chemicon/Millipore, Billerica, MA, USA, mAB374, 1:12000000 dilution) and XIAP (BD Transduction Laboratories, San Jose, CA, USA # 610716, 1:1000 dilution) were also used. Afterwards, membranes were incubated for 1 h at room temperature with anti-mouse horseradish peroxidase-conjugated secondary antibody (dilution 1:30000) (Calbiochem, La Jolla, CA, USA). Blots were developed with immobilon chemiluminescent HRP substrate (Millipore, Bedford, MA, USA) and images were captured by a FluorChem SP system (Alpha Innotech, San Leandro, CA, USA). Quantity analysis is based on the intensity of the band using the Quantity One software (Bio-Rad Laboratories, Hercules, CA, USA). The linearity of the assay was preliminarily checked for each monoclonal antibody by submitting different amounts of untreated cell extracts to western blotting.

### Statistical analysis

Results were analyzed by one-way ANOVA or by repeated-measures ANOVA using a Sidak test as a post-test. P-values below 0.05 were considered statistically significant. All data are described as the mean ± standard error (SE). All observations were confirmed by at least three independent experiments.

## Results

### Cytotoxicity studies

PE5 is cytotoxic for NCI/ADR-RES and HeLa cell lines using the MTT assay. For comparison, this experiment was also performed with onconase. The results indicated that PE5 was seven- to ten-fold less cytotoxic than onconase in both cases (Table 1). We also examined the cytotoxic effect of these RNases on N1 normal human fibroblasts. Again, both RNases were cytotoxic for N1 fibroblasts. However, in this case the difference in IC_50_s between both RNases was increased to nearly 20-fold (Table 1).

**Table 1:** IC_50 _values for PE5 and onconase for the indicated cell lines

Cell line	PE5 (μM)	Onconase (μM)
HeLa	8.2 ± 0.6	0.8 ± 0.1
NCI/ADR-RES	6.9 ± 0.8	1.1 ± 0.1
N1	19.5 ± 1.4	1.0 ± 0.2

Data are presented as mean ± SE of at least three independent experiments made in triplicates.

**Figure 1:**
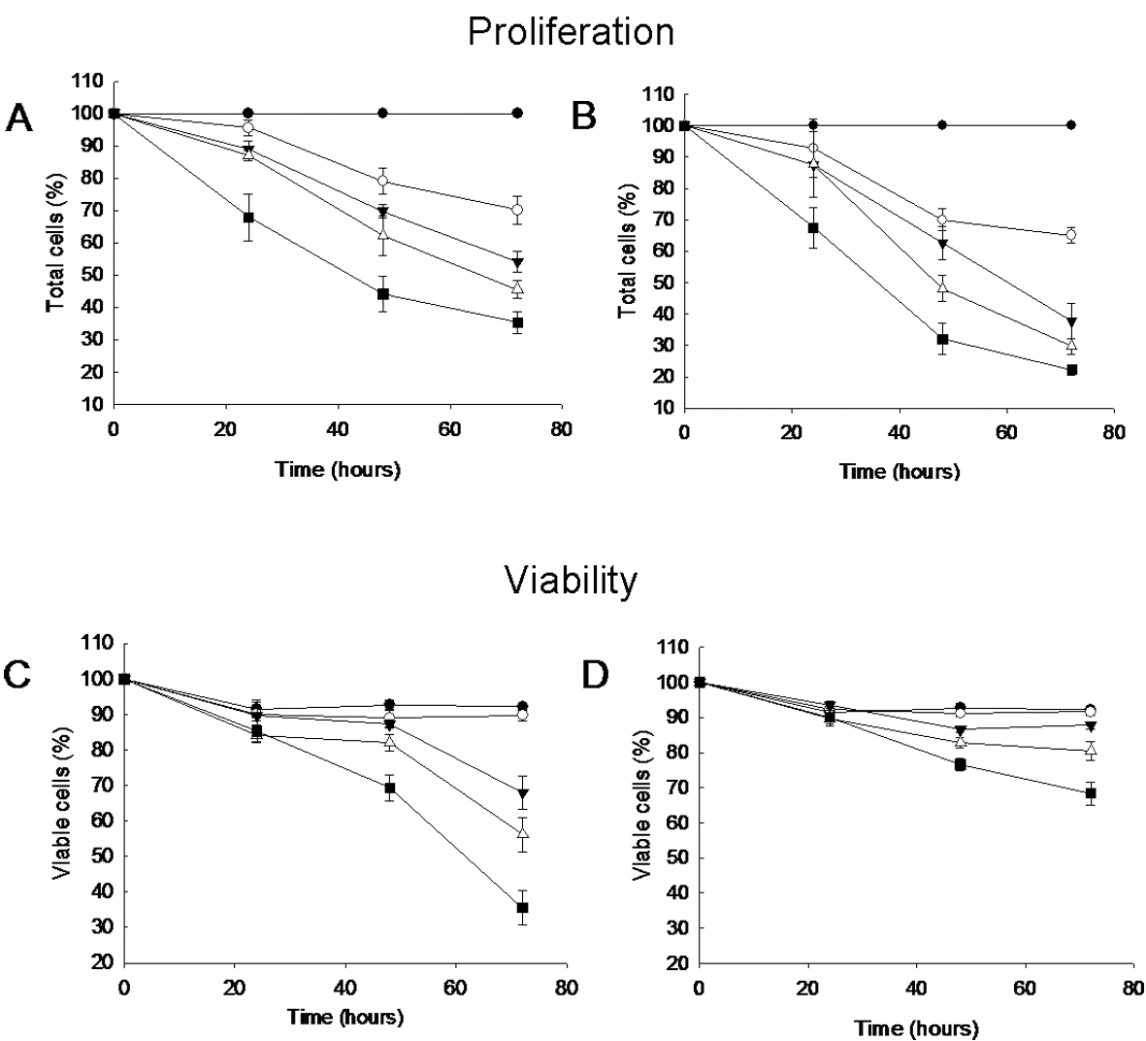
**Proliferation and viability curves of NCI/ADR-RES cells treated with PE5 or onconase**. Cells were treated with 0 (black circle), 2 (white circle), 7 (black triangle), 14 (white triangle) and 35 μM (black square) of PE5 (A and C) or 0 (black circle), 0.3 (white circle), 1 (black triangle), 2 (white triangle) and 5 μM (black square) of onconase (B and D). Trypan blue dye exclusion cell viability was estimated at 0, 24, 48 and 72 h after administration of the RNases. The plotted points represent means of at least three independent experiments.

The MTT assay does not allow discerning whether the drug exerts a cytostatic or a cytotoxic effect and therefore we investigated the effect of both proteins on cell growth and cell viability. We focused this study on the NCI/ADR-RES cell line because it was the most sensitive to PE5. Figure 1 shows the effect on NCI/ADR-RES proliferation and viability of incubating four different concentrations of PE5 or onconase for up to 72 h. Even at 24 h of intoxication an effect of both RNases on proliferation could be observed at the highest concentrations assayed. At this same time the viability of NCI/ADR-RES cells was reduced only by treatment with 14 or 35 μM PE5. Consistently with the cytotoxicity results (Table 1), cells treated with 7 μM PE5 or 1 μM onconase for 72 h (which correspond to the IC_50 _values described in Table 1) decreased the proliferation to around 50% respective to untreated cells (Figures 1A and 1B). Therefore, PE5 is mainly cytotoxic although at low concentrations a minimal cytostatic effect can be observed. On the contrary, onconase arrests proliferation at low concentrations (below to 1 μM) but begins to be cytotoxic at higher concentrations, as it has been previously described for other cell lines [2]. Interestingly, although a seven-fold lower concentration of onconase was necessary to reduce cell growth to 50%, the percentage of viable cells at each incubation time was similar when using either PE5 or onconase. This shows that both RNases induce cell death at the same extent but that onconase presents an additional capacity of arresting cell proliferation.

### Analysis of the PE5 treatment on the cell cycle phase distribution

**Figure 2:**
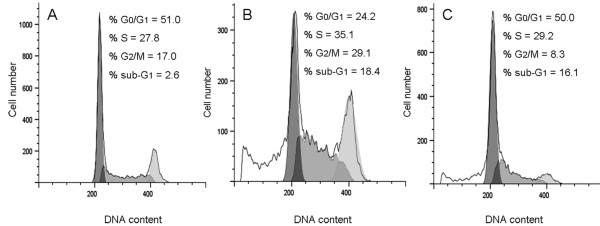
**Effects of PE5 and onconase on the NCI/ADR-RES cell cycle phase distribution after treatment for 72 h**. Untreated cells (A), 35 μM PE5 treated cells (B) and 5 μM onconase treated cells (C) were permeabilized and stained with PI. Cell DNA content was analyzed by flow cytometry. Data are representative of three independent assays. Values were analyzed from 10,000 total events.

We investigated the effect of 35 μM PE5 on the NCI/ADR-RES cell cycle progression (Figure 2). For comparison, the effect of 5 μM onconase on the cell cycle phase distribution was also investigated. After 72 h of PE5 exposure, a clear increase of S and G_2_/M cell cycle phases, concomitant with a decrement of G_0_/G_1 _cell cycle phase cells was observed. In contrast, the cell cycle phase distribution of cells treated with a cytotoxic concentration of onconase was very similar to that of untreated growing cells. In both cases, a significant proportion of RNase-treated cells were identified as a sub-G_1 _cell population which corresponds to apoptotic cells carrying fractional DNA.

**Figure 3:**
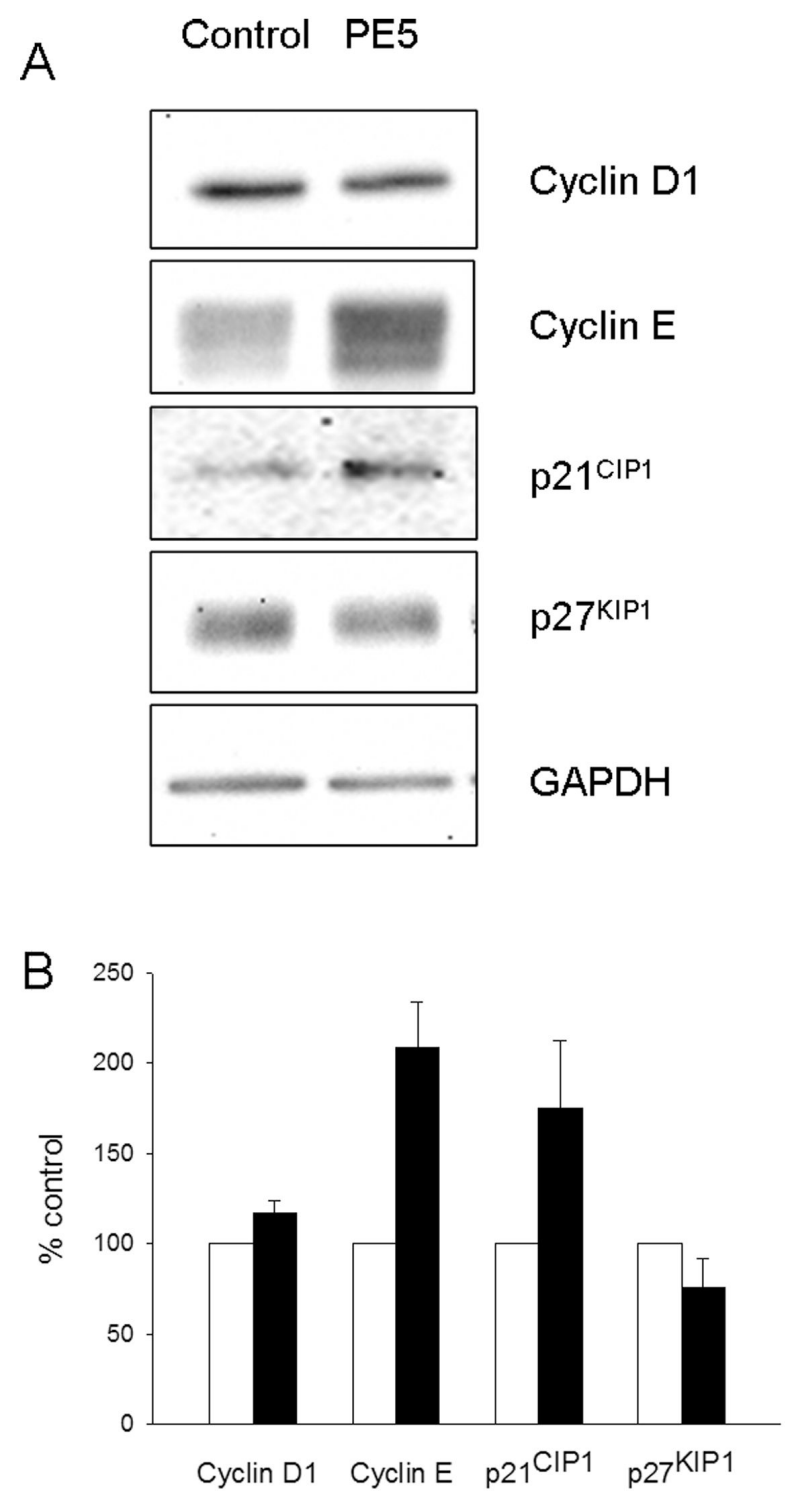
**Expression of cyclin D1, cyclin E, p21^WAF1/CIP1 ^and p27^KIP1 ^in 7 μM PE5-treated cells**. 72 h after the treatments, cells were harvested and analyzed by Western blot using the antibodies described in the Methods section. A) Western blot of a representative experiment. B) Densitometric analysis of the GAPDH-normalized immunoblots of each treatment respective to that of untreated cells. White bars indicate untreated cells (control) and black bars indicate PE5-treated cells.

To further characterize the effect of PE5 on the cell cycle phase distribution we used western blot to also analyze the expression of cyclin D1, cyclin E, p21^WAF1/CIP1 ^and p27^KIP1 ^(Figure 3). PE5 increased the expression of cyclin E to a 209% ± 25 of that of untreated cells and also that p21^WAF1/CIP1 ^to a minor extent (to 175% ± 37 of untreated cells). The amounts of cyclin D1 and of p27^KIP1 ^remained nearly unchanged respective to untreated cells.

### PE5 induces apoptosis in NCI/ADR-RES cells

**Figure 4:**
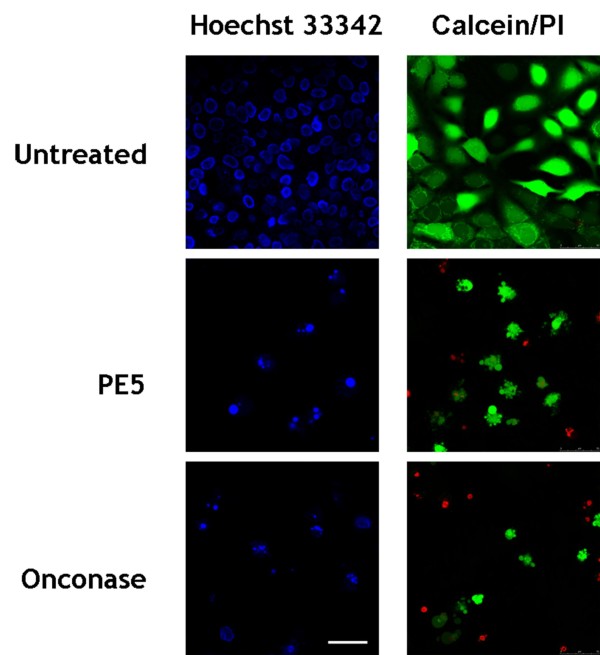
**Nuclear and cellular morphological effects of PE5 and onconase on the NCI/ADR-RES cell line**. Overnight serum-starved NCI/ADR-RES cells were treated with 35 μM PE5 or 5 μM onconase for 72 h. Subsequently, Hoechst 33342 and calcein/PI staining were performed and observed under a confocal laser microscope. Bar 50 μm.

We studied the cell death induced by the treatment with PE5 and whether the RNase-treated cells displayed characteristic features of apoptosis. Onconase was included as a control in these experiments since it has been shown that it induces apoptosis in different cell lines (for a review, see [21]). NCI/ADR-RES cells were treated with 35 μM PE5 or 5 μM onconase for 72 h, stained with Hoechst 33342 and then analyzed, using a confocal microscope. In both treatments but not in the untreated control cells, nuclear morphological changes typical of apoptosis, such as the characteristic chromatin condensation and the nuclear fragmentation, could be observed (Figure 4). The PE5 and onconase effect on NCI/ADR-RES cell morphology was also studied in fresh cultures using calcein AM and PI staining. Cells treated with 35 μM PE5 or 5 μM onconase show typical apoptosis features like membrane blebbing, apoptotic body formation, chromatin condensation and apoptotic nuclear fragmentation (Figure 4).

We sought to quantify the percentage of NCI/ADR-RES cells in early and late apoptosis after 24, 48 and 72 h of incubation with 35 μM PE5. As a control, the same experiment was performed with 5 μM onconase. The results of the FACS analysis of these cells, stained with Annexin V-Alexa Fluor 488 and PI, show that the percentage of necrosis induced by the treatment was negligible in both cases (Table 2). Again, induction of apoptosis could be demonstrated by the translocation of phosphatidylserine to the external hemi-membrane. Apoptosis was evident at 48 h of treatment in both cases and further increased at 72 h. Interestingly, the percentage of cells treated with PE5 that were at early apoptosis was nearly 50% higher than that of those treated with onconase.

**Table 2:** Apoptosis measured by Annexin V-Alexa Fluor 488/PI staining

	Control			PE5			Onconase		
	**24 h**	**48 h**	**72 h**	**24 h**	**48 h**	**72 h**	**24 h**	**48 h**	**72 h**

Early apoptotic cells (%)	4.1 ± 0.2	4.2 ± 0.5	4.5 ± 0.7	3.9 ± 0.1	17.7 ± 1.9	28.3 ± 1.3	6.8 ± 2.1	11.9 ± 1.1	20.4 ± 2.0
Late apoptotic cells (%)	5.9 ± 0.2	7.2 ± 0.9	5.7 ± 0.1	0.7 ± 0.3	6.5 ± 1.4	26.0 ± 4.4	2.3 ± 0.1	11.6 ± 2.6	25.9 ± 1.4
Necrotic cells (%)	2.4 ± 0.3	2.4 ± 0.7	1.6 ± 0.2	0.0 ± 0.0	0.0 ± 0.0	0.0 ± 0.0	0.0 ± 0.0	0.0 ± 0.0	0.0 ± 0.0
Viable cells (%)	87.6 ± 0.6	86.2 ± 1.1	88.2 ± 1.0	95.4 ± 0.4	75.8 ± 0.5	45.7 ± 5.3	90.9 ± 2.0	76.5 ± 3.7	53.7 ± 0.6

NCI/ADR-RES cells were treated with 35 μM PE5 or 5 μM onconase for 24, 48 and 72 h, stained with Annexin V-Alexa 488 and PI and analyzed by flow cytometry. Cells undergoing early apoptosis were positive for Annexin V and negative for PI (Annexin V+/PI-), late apoptotic cells were Annexin V+/PI+ and necrotic cells were Annexin V-/PI+. All data were expressed as mean ± SE of three different experiments. Values were analyzed from 10,000 total events.

### The mechanisms of apoptosis of PE5 and onconase are different

**Figure 5:**
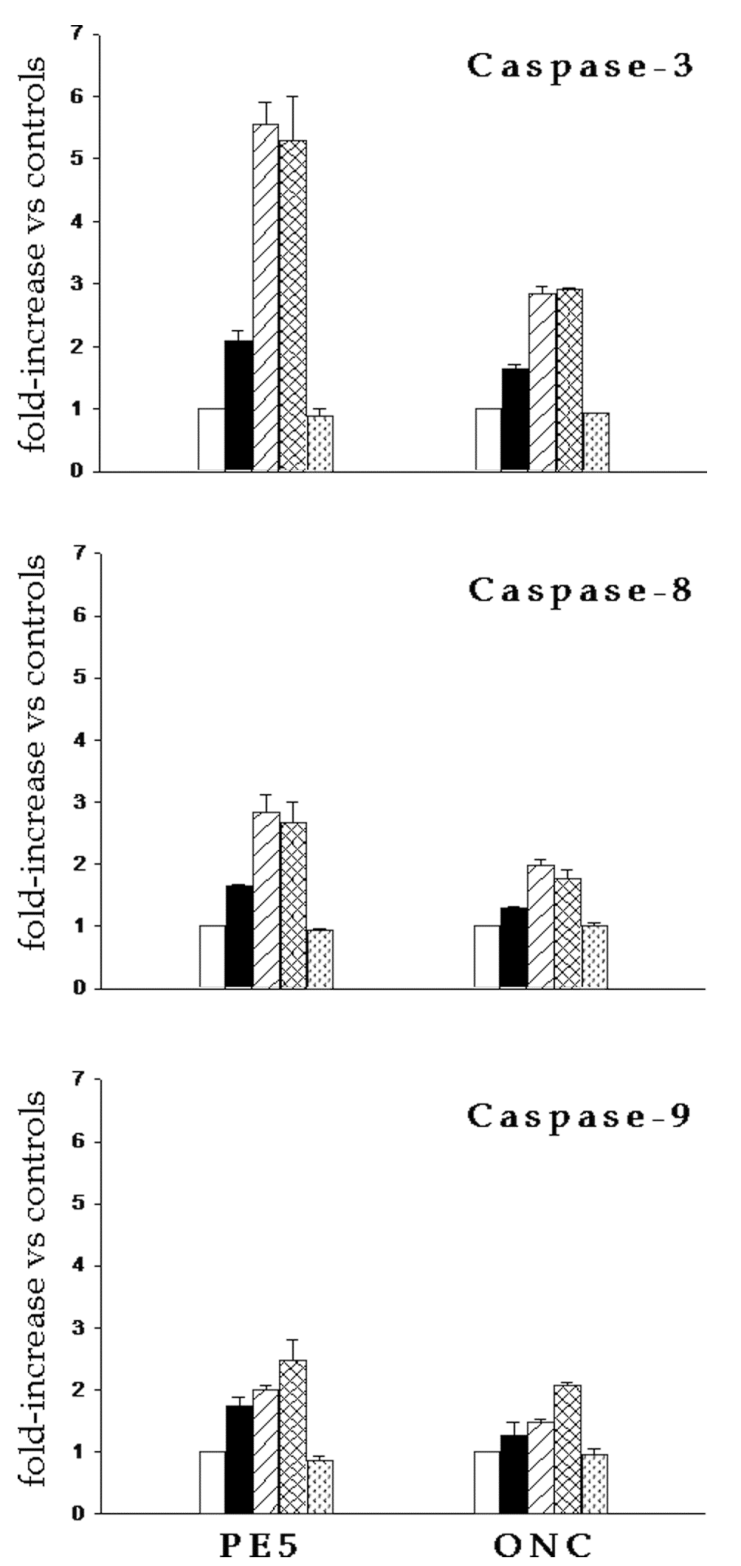
**Caspase-3, -8 and -9 activities in NCI/ADR-RES cells treated with PE5 or onconase**. Cells were treated with 35 μM PE5 or 5 μM onconase (ONC) for 24 (black bars), 48 (striped bars) and 72 h (checkered bars). White bars indicate untreated cells (control). Parallel samples were treated with the specific inhibitors of caspase-3 (z-DEVD-FMK), caspase-8 (z-IETD-FMK), caspase-9 (z-LEHD-FMK) (stippled bars) as a negative control. Activation of caspase-3, -8 and -9 was quantified in whole cell lysates using a quantitative colorimetric assay as described in the text. Results are expressed as the mean ± SE of three independent experiments.

We were interested in investigating whether the cytotoxic mechanism of both RNases could be different. In order to characterize apoptosis of NCI/ADR-RES cells induced by PE5 and onconase, activation of procaspases-3, -8 and -9 was investigated (Figure 5). PE5 induces the activation of the procaspases-3 and -8 even at 24 h of drug incubation (p < 0.05). Activation reaches its maximum at 48 h. Activation of procaspase-9 is also induced by PE5 but it can be only detected after 48 h of incubation with the RNase and continues to increase at 72 h. For onconase, procaspase-8 and -9 activation was delayed to 48 h of RNase incubation (p < 0.05). The pattern of activation is very similar to that found for PE5. At 72 h of incubation with onconase the three caspases were active although to a minor extent when compared to PE5 treatment. Caspase activity was also investigated after 72 h of RNase incubation in the presence of the caspase-specific inhibitors DEVD-FMK, IETD-FMK or LEHD-FMK. In the presence of these inhibitors the activity dropped to the level of the control extracts.

We investigated by western blot the effect produced by PE5 on the level of different apoptosis-related proteins. Cellular extracts from NCI/ADR-RES treated for 72 h with 7 μM PE5 were subjected to immunoblotting using antibodies against Bcl-2, Bax, XIAP, JNK and the phosphorylated form of p46JNK. For comparison, additional experiment incubating NCI/ADR-RES with 5 μM onconase were performed.

**Figure 6:**
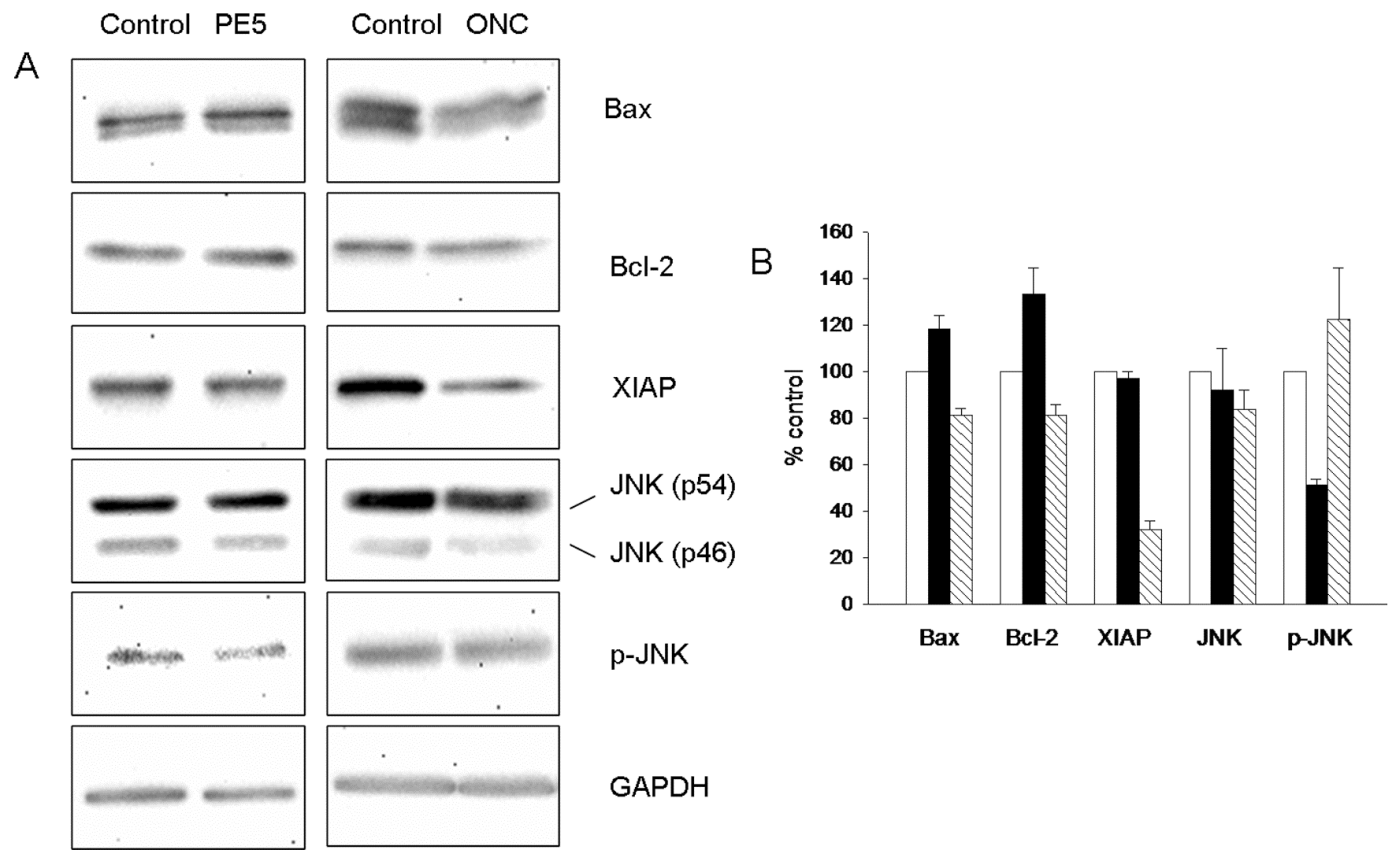
**Effects of 7 μM PE5 or 5 μM onconase on the expression of Bcl-2, Bax, JNK and XIAP and on the phosphorylation of p46 JNK in NCI/ADR-RES after treatment for 72 h**. Protein levels were determined by Western blot analysis (see Methods section). A) Western blot of a representative experiment. B) Densitometric analysis of the GAPDH-normalized immunoblots of each treatment respective to that of untreated cells. JNK and p-JNK refers to the unphosphorylated and phosphorylated p46 JNK isoforms, respectively. White bars indicate untreated cells (control) and black bars and striped bars indicate PE5- and onconase-treated cells, respectively.

We show in Figure 6 the effects of the treatment of NCI/ADR-RES with either PE5 or onconase on the expression of these apoptotic-related proteins. PE5 did not change the expression levels of any of the assayed proteins. However, it induced a clear decrease of the level of the phosphorylated form of p46 JNK respective to untreated cells (two-fold decrease). For onconase, important differences could be detected when compared with PE5. In this case, the levels of Bax, Bcl-2 and JNK remained unaltered compared to untreated cells but a clear decrease of XIAP was observed. On the other hand, we could not detect changes in the levels of phosphorylated form of JNK respective to untreated cells. Although JNK has been implicated largely in stress-induced apoptosis it has been postulated that JNK exerts an anti-apoptotic activity in p53-deficient tumor cells [22]. We therefore tested whether SP600125, a known inhibitor of JNK [23], could produce a cytotoxic effect on NCI/ADR-RES cells. As expected, SP6000125 was cytotoxic to NCI/ADR-RES cells being its IC_50 _after 96 h of exposure of 56.3 ± 2.1 μM (results not shown).

## Discussion

Although HeLa cell line has wild type p53 alleles, it has been reported [24] that it contains no detectable p53 tumor suppressor protein. NCI/ADR-RES contains mutated p53 and, unlike the HeLa cell line, overexpresses P-gp and GST-π multidrug resistance proteins [25-27] and also XIAP and survivin anti-apoptotic proteins [28]. Both cell lines were similarly sensitive to PE5 showing that the cytotoxicity of this RNase is not prevented by a mutated p53 or multidrug resistance phenotypes. Both cell lines were also very affected by onconase. This was expected since it was previously reported that onconase induces cell death in other MDR cell lines and that the proapoptotic activity of p53 is not required for onconase-induced apoptosis [29].

We observed that N1 cells treated with PE5 were clearly less affected than those treated with onconase. This is indicative that the specificity of PE5 for tumor cells could be almost equal to that of onconase. Although *in vivo *onconase presents clear anti-tumor activity, the cytotoxicity of this RNase for non-tumor cell lines has been described to be variable. For example, Haigis and coworkers [30] reported the cytotoxicity of onconase for different tumor and non-tumor cell lines. They found that the IC_50 _of IMR-90 lung fibroblast cell line was similar to those of tumor cell lines whereas the IC_50 _of WI-38 lung fibroblasts increased ten-fold.

PE5-treated cells display classical hallmarks of apoptosis. This is very interesting since a non-apoptotic mode of cell death could hamper the potential application of PE5 as an anticancer drug. As expected, onconase also induces the apoptosis in NCI/ADR-RES, as it has been previously reported for different cell lines (for a review, see [4]). Both RNases induce the activation of initiation of procaspases-8 and -9 which lead to the activation of executioner procaspase 3 in NCI/ADR-RES cells. Furthermore, no changes in the proportion of Bcl-2 amounts respective to those of Bax are detected in both cases. These results suggest that the treatment of NCI/ADR-RES with PE5 or onconase induces both extrinsic and intrinsic apoptotic pathways, the latter being independent of Bcl-2 and Bax. The mode by which onconase induces cell death has been shown to be dependent on the cell line used in the study. Onconase has been shown to induce apoptosis by activating caspases and serine proteases in some cases [29,31] but, recently, onconase-induced caspase-independent death of neuroblastoma cell lines have been reported [32]. Although processing of procaspases-9, -3, and -7 but not of procaspase-8 has been described [29], it is not clear to what extent the mitochondrial apoptotic pathway is involved since no cytochrome C release or sustained decrease of Bax has been observed [29]. In contrast, for the HL-60 human promyelocytic leukemia cell line, onconase induced the expression of Bax while decreasing that of Bcl-2 [33].

During apoptosis cell cycle progression is stopped [34]. The FACSCalibur flow cytometric analysis shows that cell death induced by PE5 is accompanied by an accumulation of cells in the S- and G_2_/M-phases in the NCI/ADR-RES cell line. This may indicate that PE5 arrest cell cycle at S- and G_2_/M-phases or, alternatively, that there is preferential PE5-induced cell death in G_1 _cell-cycle phase, which leads a proportionally higher percentage of S and G_2_/M. Over-expression of cyclin E and p21^WAF1/CIP1 ^explains the observed reduction of the percentage of cells in G_0_/G_1 _cell-cycle phase together with the increase of the percentage of cells in S and G_2_/M cell-cycle phase. Cyclin E is a G_1 _cyclin expressed near the G_1 _to S transition and drives entry into the S phase by binding to CDK2. Increase of cyclin E after S or G2/M arrest has been already observed in different works [35,36]. p21^WAF1/CIP1 ^acts as a dual inhibitor of cyclin-dependent kinases (CDKs) [37] and proliferating-cell nuclear antigen [38]. As a cyclin-dependent kinase inhibitor, p21^WAF1/CIP1 ^interacts with several cyclins and CDKs leading to inhibition of these kinases with subsequent cell cycle arrest. Overexpression of p21^WAF1/CIP1 ^results in G_1_-, G_2_- [39] or S-phase arrest [40]. Interestingly, it has been described [39] that the presence of functional pRb correlates with preponderant G_1 _arrest in response to overexpression of p21^WAF1/CIP1 ^whereas G_2 _arrest is more prominent in pRb-negative cells, as is the case of NCI/ADR-RES [41]. As a proliferation inhibitor, p21^WAF1/CIP1 ^is poised to play an important role in preventing tumor development. For example, it has been show that celecoxib leads to up-regulation of p21^WAF1/CIP1 ^along with down-regulation of cyclin B1 promoting G_2_/M arrest and induction of apoptosis in a p53-independent pathway [42]. Histone deacetylase inhibitor trichostatin A induces p21^WAF1/CIP1^, cell cycle arrest, and apoptosis in human gastric and oral carcinoma cell lines [43]. Pioglitazone, a peroxisome proliferator-activated receptor-γ agonist, induced p21^WAF1/CIP1 ^and enhanced the sensitivity of carcinoid cells to apoptosis induced by the TRAIL ligand. Adenovirus-mediated overexpression of p21^WAF1/CIP1 ^in these cells significantly enhanced the level of TRAIL-induced apoptosis [44]. Overexpression of p21^WAF1/CIP1 ^in the p53-deficient human ovarian carcinoma cell lines SKOV3 and OVCAR3 also led to increased apoptosis in response to cisplatin treatment [45].

Our results suggest that the apoptosis induced by PE5 is mediated through p21^WAF1/CIP1 ^and JNK. It has been shown that p21^WAF1/CIP1 ^acts as a non-enzymatic inhibitor of stress activated protein kinases (SAPKs)/JNKs [46]. Accordingly, JNK is underphosphorylated in PE5-treated cells. Although JNK has been implicated largely in stress-induced apoptosis, under some circumstances, JNK plays a protective role and supports cell survival, and evidence is accumulating that JNK plays a role in cell proliferation, cell transformation and tumor progression[22]. The anti-apoptotic function of JNK has been related to the status of p53, exerting its anti-apoptotic activity in p53-deficient tumor cells [22]. The p21^WAF1/CIP1 ^mediated inhibition of JNK in PE5 treated cells would promote the apoptosis of NCI/ADR-RES cells. Similar examples of induction of apoptosis by JNK inhibition have been previously reported. For example, treatment with antisense oligonucleotides to JNK2 and, to a lesser extent, to JNK1, suppresses growth and induces apoptosis of tumor cell lines with mutant but not wild-type p53 [47]. It has been also reported that the pharmacological inhibition of JNK using SP600125 is associated with an increase in the G_2_/M cell-cycle phase and apoptosis in different cell lines [48,49] being some of them (MCF-7 and Sk-Br-3) sensitive to PE5 (Castro et al., unpublished data). Finally, it has been shown [50] that cells immortalized via retrovirus-mediated transfer of E6 HPV gene product, which targets p53 for degradation, are more sensitive to onconase when both JNK1 and JNK2 alleles are disrupted. Accordingly, we also show that the pharmacological inhibition of JNK with SP600125 also produces a cytotoxic effect in NCI/ADR-RES cells.

Regarding onconase, we show that it does not arrest the proliferation at a defined cell cycle phase. Although a general consensus in the literature indicates that onconase arrests proliferation at the G_0_/G_1 _cell cycle phase [3], this is far from always being the rule. Onconase arrests NIH/3T3 cells at the G_2_/M cell cycle phase [51], but for Jurkat cells the arrest of proliferation induced by onconase could not be attributed to alterations in the cell cycle phase progression [52].

The effects of PE5 on NCI/ADR-RES cells are clearly different to those induced by the treatment with onconase. The cell cycle phase distribution and the proportion of cells at early and late apoptosis are very different. On the other hand, treatment with onconase decreases three-fold the concentration of XIAP respective to untreated cells whereas it does not change the level of the phosphorylated form of JNK. In this sense, onconase and PE5 seem to be bypassing different mechanisms of survival, mediated by XIAP and JNK, respectively. These results are very indicative that both RNases kill the cells through different mechanisms of apoptosis. The different mechanisms are likely related to their mechanism of action. Onconase is driven into the cytoplasm and its natural substrates are t-RNA and most probably the non-coding RNA (microRNA) [53] whereas PE5 is located in the nucleolus [10] and targets nuclear RNA [12]. Interestingly, nucleolus and biogenesis of rRNA are important factors distinguishing cancer from normal cells and may have an active role in tumorigenesis [54]. Altogether, makes the use of both RNases in combination as chemotherapeutical agents very attractive.

## Conclusions

We have demonstrated that PE5 does not arrest cell proliferation but rather induces cell death by apoptosis. We show that the cytotoxicity of PE5 is associated by the increase of the expression of p21^WAF1/CIP1 ^together with the underphosphorylation of JNK. We also show that the mechanism of cytotoxicity of PE5 is different to that induced by the onconase. *In vivo *experiments with PE5 are warranted in order to evaluate its anticancer capacity.

## Competing interests

The authors declare that they have no competing interests.

## Authors' contributions

JC participated in the design of the study, carried out all the experiments. MR participated in the design of the study and revised the manuscript critically. SN and MVN were involved in the laser scanning confocal microscope experiments, interpretation of data and manuscript revising. MV and AB participated in the design of the study, interpretation of data and wrote the manuscript. All authors read and approved the final manuscript.

## Pre-publication history

The pre-publication history for this paper can be accessed here:

http://www.biomedcentral.com/1471-2407/11/9/prepub
